# Light-Based Therapies for Metabolic Modulation of Mesenchymal Stem Cells in Regenerative Dentistry

**DOI:** 10.1007/s10103-026-05008-x

**Published:** 2026-09-08

**Authors:** Ygor Gonçalves Felix de Mattos, Otávio Augusto Alfonso Morais de Melo, José Ricardo Muniz Ferreira, Jamil Awad Shibli, Letícia Mello Bezinelli, Fernanda de Paula Eduardo, Alessandra V. S. Faria, Daniela Franco Bueno

**Affiliations:** 1 Faculdade Israelita de Ciências da Saúde Albert Einstein, Hospital Israelita Albert Einstein, São Paulo, Brazil; 2 RCRIO, Campinas, Brazil; 3 Institute of Biomedical Sciences, Universidade de São Paulo, São Paulo, Brazil

**Keywords:** Mesenchymal stem cells (MSCs), Photobiomodulation (PBM), Low-level laser therapy, Regenerative dentistry, Mitochondrial metabolism, Osteogenesis, adipogenesis, chondrogenesis, angiogenesis, Light-Based Therapies

## Abstract

Periodontal diseases and temporomandibular disorders are characterized by chronic inflammation and progressive destruction of supporting tissues, leading to functional and aesthetic impairments. In this context, regenerative dentistry has increasingly focused on minimally invasive strategies capable of restoring tissue structure and function. Mesenchymal stem cells (MSCs) have emerged as central elements of regenerative approaches due to their multilineage differentiation potential, immunomodulatory properties, and paracrine activity. Concurrently, low-level laser therapy (LLLT), also known as photobiomodulation (PBM), has gained relevance as a non-invasive modality capable of modulating cellular behavior, inflammation, and tissue repair. This scoping review synthesizes current evidence on the combined application of MSCs and light-based therapies, emphasizing how laser-mediated metabolic modulation influences MSC fate and regenerative outcomes. Overall, the findings demonstrate that PBM enhances MSC proliferation, viability, migration, and lineage-specific differentiation, osteogenic, chondrogenic, angiogenic, and adipogenic, in a wavelength-, energy-, and dose-dependent manner. Emerging evidence indicates that the mitochondrial function represents a critical mechanistic link between laser irradiation and MSC responses. Photobiomodulation modulates mitochondrial bioenergetics primarily through activation of cytochrome c oxidase, resulting in increased ATP production, controlled reactive oxygen species signaling, and regulation of key transcription factors such as RUNX2, Sox9, and PPARγ. These mitochondrial-mediated effects act as metabolic checkpoints that integrate microenvironmental cues with lineage commitment and tissue-specific regeneration. Collectively, the data support the concept that precise modulation of mitochondrial activity by PBM optimizes the regenerative potential of MSCs. A deeper understanding of the interplay between laser strategies and parameters, mitochondrial metabolism, MSC source, and the cellular microenvironment is essential for the development of safe, effective, and reproducible regenerative protocols in dentistry and regenerative medicine.

## Introduction

Periodontal disease and temporomandibular joint disorders are highly prevalent conditions characterized by chronic inflammation and progressive destruction of supporting tissues, often leading to functional impairment, pain, and aesthetic dissatisfaction. These conditions negatively affect mastication, speech, swallowing, and sleep, resulting in significant physical and psychological consequences for affected individuals [1]. Although conventional therapeutic approaches are effective in controlling inflammation and disease progression, they frequently fail to fully restore the lost tissues, underscoring the need for regenerative strategies capable of reestablishing both structure and function.

In this context, regenerative medicine has expanded its scope in dentistry, focusing on minimally invasive approaches that promote tissue repair while reducing postoperative morbidity and optimizing functional outcomes. Among the available regenerative tools, mesenchymal stem cells (MSCs) have emerged as a central component of tissue engineering strategies due to their multipotent differentiation capacity, immunomodulatory properties, and paracrine activity [2]. These characteristics make MSCs particularly attractive for applications in periodontal regeneration, alveolar bone repair, cartilage reconstruction, and temporomandibular joint therapies.

Despite their promising biological potential, the regenerative efficacy of MSCs is highly dependent on the surrounding microenvironment. Factors such as inflammatory mediators, oxygen tension, mechanical stimuli, and biochemical signaling critically influence MSC proliferation, migration, differentiation, and secretory behavior [3]. Therefore, strategies capable of modulating MSC responses in a controlled and predictable manner have become a major focus of current research. Physical stimuli, including photodynamic and photobiomodulatory approaches, have been investigated as non-invasive tools to influence cellular activity and promote tissue homeostasis through adaptive biological responses, a phenomenon often described as the hormetic effect [4].

Low-level laser therapy (LLLT), also known as photobiomodulation, has gained increasing relevance in dentistry due to its ability to interact with biological tissues and modulate cellular metabolism, inflammatory responses, and healing processes [5]. When applied under appropriate parameters, LLLT has been shown to enhance collagen synthesis, stimulate angiogenesis, and influence stem cell behavior without inducing thermal damage. In this context, the association between MSC-based therapies and LLLT represents a promising strategy to optimize regenerative outcomes by modulating cellular activity at multiple biological levels.

Given the growing interest in combining MSCs with light-based photobiomodulation, this scoping review aims to synthesize current evidence regarding the effects of light-based strategies on mesenchymal stem cell behavior and differentiation. Emphasis is placed on osteogenic, chondrogenic, angiogenic, and adipogenic outcomes relevant to regenerative dentistry, highlighting biological mechanisms involved in these processes while addressing current challenges and future perspectives.

## Inclusion and Exclusion Criteria

This scoping review considered eligible studies directly related to all light-based photobiomodulation strategies for inclusion. In vitro studies with an exclusive focus on the production of bone, cartilage, vascular and adipose tissue lineages were included. In vivo studies involving light-based strategies applied to cells derived from the oral cavity or commonly used in angiogenesis and adipogenesis (Dental Pulp Stem Cells (DPSCs), Periodontal Ligament Stem Cells (PDLSCs), and Stem Cells from the Apical Papilla (SCAPs), Human adipose derived stem cells (ADSCs), Bone marrow stem cells (BMSCs) e Human Umbilical Vein Endothelial Cell (HUVECs) were also considered.

Studies involving human umbilical vein endothelial cells were included due to the fundamental role of angiogenesis in tissue regeneration and its well-established association with MSC-mediated regenerative mechanisms.

Studies not related to dental research, and in vivo studies conducted outside the dental field, were used only for contextualization and to demonstrate research within the field of medicine. Case reports, short communications, and letters to the editor were not included.

## Methods

The proposed scoping review was conducted in accordance with the Joana Bricks Institute (JBI) methodology for scoping reviews and with the Preferred Reporting Items for Systematic Reviews and Meta-Analyses Extension for Scoping Reviews (PRISMA-ScR) guidelines.

It is noteworthy that this review has limitations related to the availability of articles. Among these, the absence of in vivo studies focusing on adipose tissue formation in the context of dentistry is evident. Furthermore, although low-level laser is currently gaining attention, there remains a lack of studies establishing standardized protocols for its application in in vitro research.

## Search strategy

Searches were conducted in the MEDLINE database via PubMed and in LILACS, selected for their relevance and comprehensive coverage of the Health Sciences literature. The choice of these databases was intended to encompass both the international biomedical literature (MEDLINE - Pubmed) and scientific production from Latin America and the Caribbean (LILACS), ensuring a broad and representative retrieval of the available evidence on the topic.

A three-step search approach was adopted for this review. First, a preliminary and limited search was conducted in the selected databases to identify relevant articles. Subsequently, the terms contained in the titles and abstracts of the retrieved studies, as well as the index terms used to describe them, were analyzed and employed in the development of a comprehensive search strategy for MEDLINE (via PubMed). Finally, this strategy was adapted for the LILACS database, considering the specific characteristics and structure of the controlled vocabularies Medical Subject Headings (MeSH) and Health Sciences Descriptors (DeCS).

In MEDLINE (Pubmed) database - *only method of description* - Low-Level Light Therapy” OR photobiomodulation OR Low-level laser therapy OR LLLT AND Mesenchymal Stem Cells OR mesenchymal stem cells OR dental pulp stem cells OR DPSCs OR periodontal ligament stem cells OR PDLSCs OR stem cells from the apical papilla OR SCAP AND.

Cell Differentiation OR angiogenesis OR osteogenesis OR adipogenesis OR chondrogenesis.

In LILACS database – *First research method* – Field: Photobiomodulation OR Low-Level Laser Therapy OR Low-Level Light Therapy AND Mesenchymal Stem Cells OR Dental Pulp Stem Cells OR Periodontal Ligament Stem Cells OR Stem Cells from the Apical Papilla AND Cell Differentiation OR Angiogenesis OR Osteogenesis OR Adipogenesis OR Chondrogenesis.

In LILACS database – *Second research method* – Field 1: Photobiomodulation OR Low-Level Laser Therapy AND Mesenchymal Stem Cells OR Dental Pulp Stem Cells OR.

Periodontal Ligament Stem Cells OR Stem Cells from the Apical Papilla.

It is important to note that the search strategy employed in MEDLINE (via PubMed) was selected because of its ability to provide comprehensive coverage of the available evidence. In the LILACS database, two descriptor-based search strategies were applied. The first strategy was designed to be more specific to the scope review topic, whereas the second employed broader descriptors to maximize the retrieval of potentially relevant studies and provide a more comprehensive assessment of the literature.

**Fig. 1:**
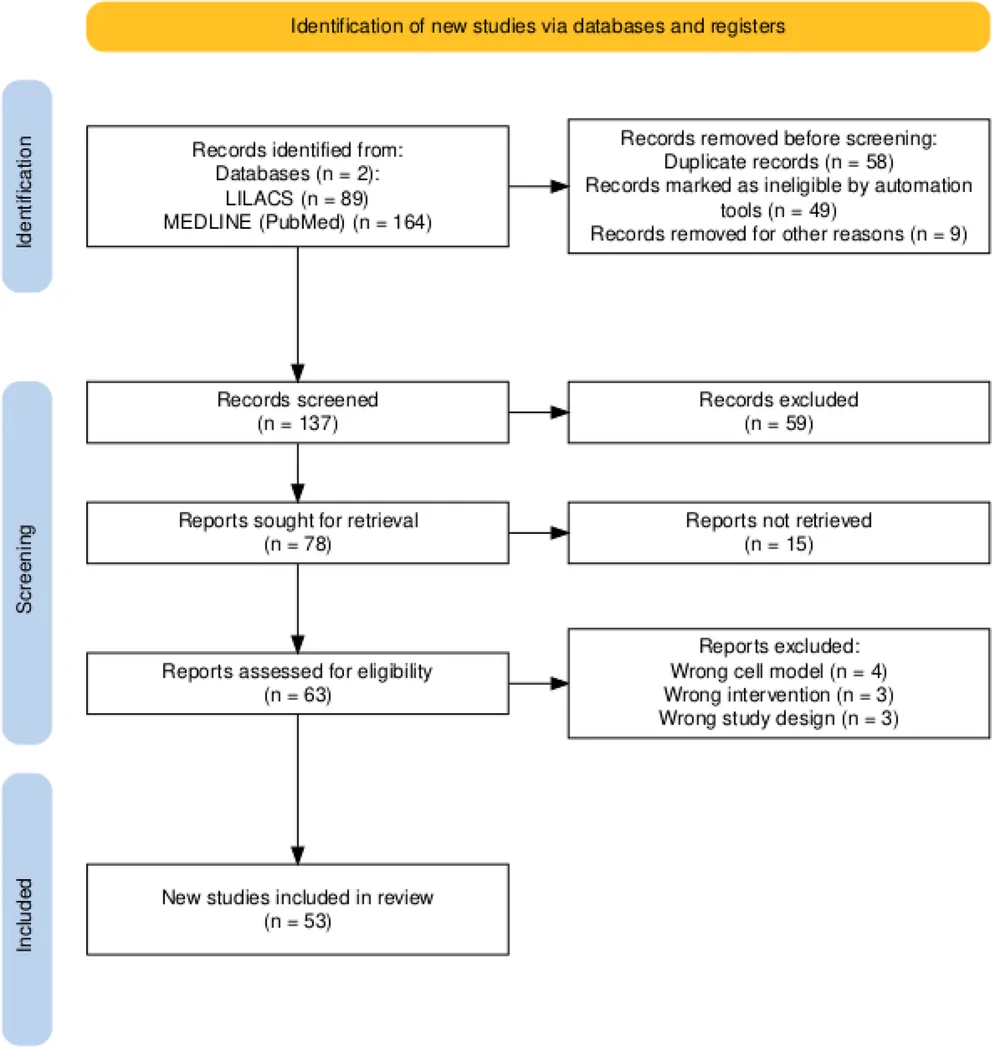
PRISMA-ScR flow diagram illustrating the process of identification, screening, eligibility assessment, and inclusion of studies in this scoping review

The identified studies were imported into Rayyan (www.rayyan.ai*)* for duplicate removal and screening according to the predefined eligibility criteria. Data collection from the MEDLINE (PubMed) and LILACS databases was performed independently by two reviewers (YFM and OAM). Any disagreements were resolved through consultation with a third reviewer (DFB). This scoping review was conducted and reported in accordance with the Preferred Reporting Items for Systematic Reviews and Meta-Analyses Extension for Scoping Reviews (PRISMA-ScR) guidelines and is detailed in the PRISMA-ScR flow diagram (Fig. 1).

### Mesenchymal stem cells as central effectors of tissue regeneration in dentistry

MSCs are multipotent stromal cells that play a fundamental role in tissue maintenance and regeneration. These cells can be isolated from a wide range of adult and perinatal tissues, including bone marrow, adipose tissue, dental pulp, periodontal ligament, gingiva, and exfoliated deciduous teeth [6, 7]. According to established criteria, MSCs exhibit adherence to plastic under standard culture conditions, expression of specific surface markers, and the ability to differentiate into osteogenic, chondrogenic, and adipogenic lineages when exposed to appropriate stimuli [2].

Stem cells are commonly classified based on their differentiation potential, which progressively decreases across categories. Totipotent cells, such as the zygote, possess the ability to generate all embryonic and extraembryonic tissues. Pluripotent stem cells (PSCs), derived from the inner cell mass of the blastocyst, can form all somatic cell types but do not contribute to extraembryonic structures [8]. In contrast, MSCs are classified as multipotent cells, with the capacity to differentiate into multiple cell types within the mesodermal lineage. This restricted yet versatile differentiation potential, combined with fewer ethical concerns and a lower risk of tumorigenicity compared to PSCs makes MSCs particularly suitable for translational and clinical applications in dentistry [8].

Beyond their differentiation capacity, MSCs exert significant paracrine and immunomodulatory effects. Through the secretion of cytokines, growth factors, and extracellular vesicles, MSCs regulate inflammation, angiogenesis, and tissue repair, thereby contributing to regenerative processes even when direct differentiation into target tissues is limited. These properties have positioned MSCs as promising candidates for cell-based therapies in periodontal regeneration, maxillofacial reconstruction, and temporomandibular joint disorders [2].

An important advantage of MSCs in dental and craniofacial applications lies in the accessibility of oral tissues as cell sources. Stem cells derived from the pulp of deciduous teeth, for example, can be obtained through minimally invasive procedures and exhibit high proliferative potential and robust differentiation capacity. When isolated and characterized according to the guidelines established by the International Society for Cell and Gene Therapy, these cells demonstrate significant potential for use in tissue engineering and regenerative therapies [3]. 

However, the biological behavior of MSCs is not intrinsic or static; rather, it is strongly influenced by external stimuli and microenvironmental conditions. Chemical, mechanical, and physical factors can modulate MSC proliferation, differentiation pathways, and functional profiles. Among these modulatory factors, physical stimuli such as light-based therapies have gained attention for their ability to influence cellular activity in a controlled and non-invasive manner. Photobiomodulation has been shown to affect MSC responses by modulating intracellular signaling pathways and metabolic activity, thereby contributing to tissue homeostasis and regenerative balance [9].

In summary, mesenchymal stem cells represent a cornerstone of regenerative strategies in dentistry due to their multipotency, immunomodulatory capacity, and accessibility from dental tissues. A deeper understanding of how external modulatory approaches, such as LLLT, interact with MSC biology, is essential for optimizing regenerative protocols and advancing safe and effective clinical applications.

The current literature on photobiomodulation (PBM) frequently fails to clearly distinguish between dental-derived mesenchymal stem cells (MSCs), such as dental pulp stem cells (DPSCs) and periodontal ligament stem celldental MSCs, including adipose-derived (ADMSCs), bone marrow-derived MSCs (BMSCs) and human umbilical vein endothelial cells (HUVECs), representing a significant limitation in the interpretation and comparison of results [10], as these cell populations differ in origin, biological behavior, and response to photobiomodulation protocols [11]. 

## Photobiomodulation as a metabolic and microenvironmental modulator of MSC behavior

In dentistry and regenerative medicine, photobiomodulation (PBM) has been widely investigated for its ability to reduce pain, modulate inflammatory responses, and accelerate tissue healing. More recently, increasing attention has been directed toward its potential to influence stem cell behavior, particularly MSCs, which are central to tissue regeneration. Lasers used in photobiomodulation can be classified according to the wavelength of light emitted, including blue (400–500 nm), green (500–600 nm), red (600–700 nm), and infrared (700–1000 nm) spectra [12]. The biological effects of these wavelengths vary according to their penetration depth, energy density, and interaction with cellular components. Red and infrared wavelengths are the most extensively studied in regenerative applications, as they penetrate tissues more effectively and have demonstrated favorable effects on cell viability, proliferation, and differentiation. Red light is generally associated with superficial tissue modulation, whereas infrared light penetrates deeper structures, making it suitable for applications involving bone, joints, and muscles, as well as for enhancing blood and lymphatic circulation [13].

Although less explored, green and blue wavelengths have also been investigated in cellular models. Green laser irradiation (approximately 532 nm) has been reported to increase type I and type III procollagen expression in human skin fibroblasts [14], while blue light has demonstrated antimicrobial effects and the ability to modulate mitochondrial respiration under controlled conditions [15]. However, studies also indicate that shorter wavelengths may induce cytotoxic effects when inappropriate parameters are applied, highlighting the importance of careful dose selection to avoid cellular stress or irreversible damage [9, 15].

The biological effects of photobiomodulation on MSCs are closely related to its interaction with intracellular metabolic processes. Light-cell interactions occur primarily at the subcellular level, where photoreceptors absorb photons and trigger downstream signaling cascades. Among intracellular organelles, mitochondria play a relevant role in mediating cellular responses to PBM due to their central function in energy production and redox balance. Chromophores such as cytochrome c oxidase, a key enzyme in the mitochondrial respiratory chain, absorb light in the red and near-infrared spectra, leading to transient changes in mitochondrial membrane potential, ATP synthesis, and reactive oxygen species (ROS) signaling [9].

In the context of MSC biology, these mitochondrial responses do not act as isolated determinants but rather as part of a broader network of signaling pathways that regulate cell fate. Controlled modulation of mitochondrial activity through photobiomodulation has been associated with enhanced MSC proliferation, migration, and survival, as well as with the activation of transcription factors involved in lineage commitment. Importantly, this response follows a dose-dependent and hormetic pattern, in which low to moderate energy densities promote adaptive cellular responses, whereas excessive irradiation may disrupt redox homeostasis and impair regenerative potential [4, 9].

Therefore, the relevance of low-level lasers in regenerative dentistry lies not only in their direct effects on tissues but also in their ability to modulate MSC behavior in a controlled and non-invasive manner. By influencing intracellular metabolism, including mitochondrial bioenergetics, photobiomodulation can enhance the responsiveness of MSCs to their microenvironment and support regenerative processes such as osteogenesis, chondrogenesis, angiogenesis, and adipogenesis. Nevertheless, these effects are highly dependent on parameters such as wavelength, power output, energy density, and exposure time, emphasizing the need for standardized protocols and careful experimental design [13].

In summary, light-based therapies represent a valuable adjunctive approach in MSC-based regenerative strategies. When appropriately applied, photobiomodulation can modulate cellular metabolism and signaling pathways that support MSC function and tissue repair. Understanding the interaction between laser parameters, intracellular mechanisms, including mitochondrial activity, and the MSC microenvironment is essential for maximizing therapeutic efficacy while minimizing adverse effects.

## Emerging evidence on MSC–photobiomodulation interactions across osteogenic, chondrogenic, angiogenic, and adipogenic lineages

**Fig. 2:**
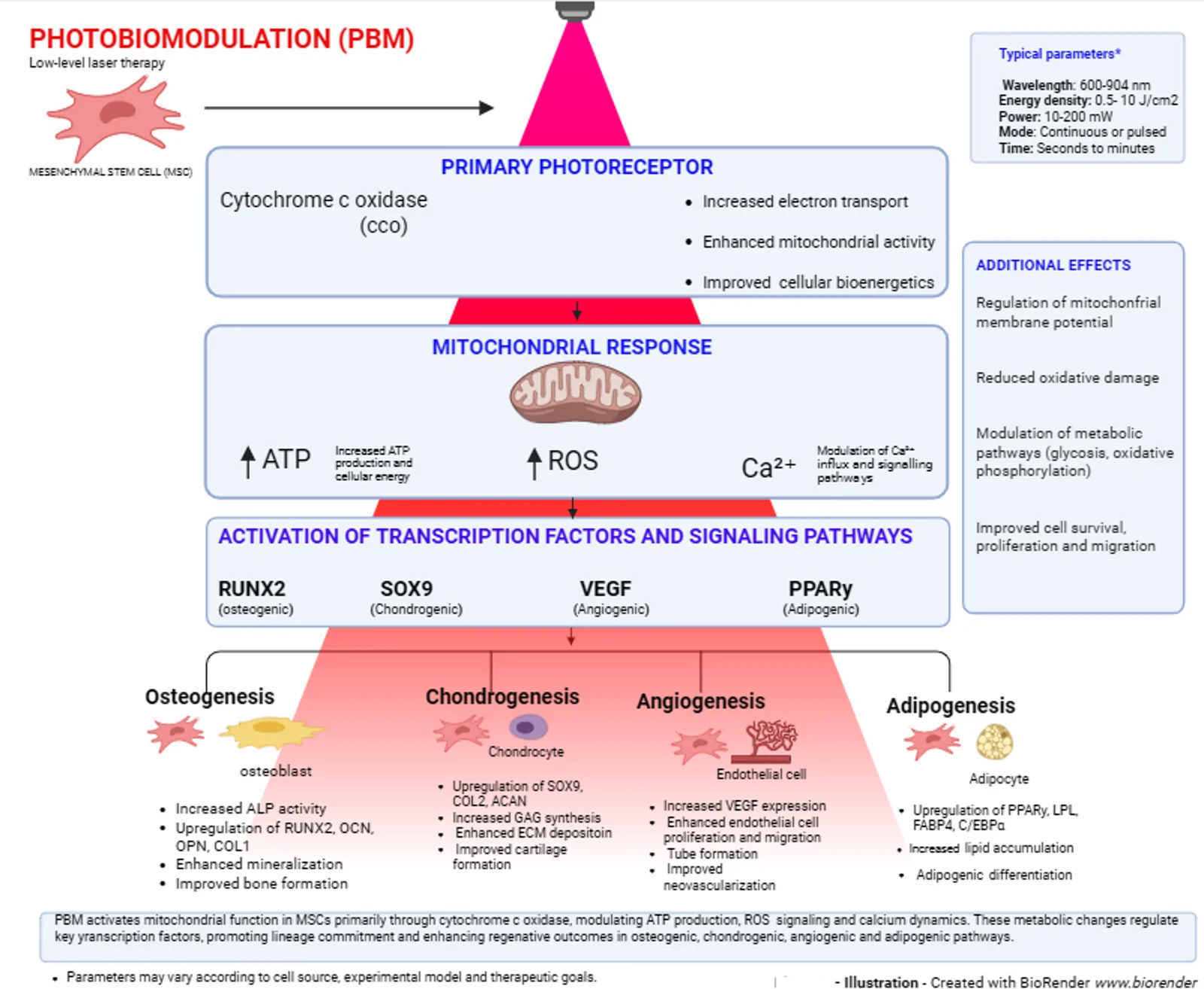
Mitochondria as metabolic integrators of photobiomodulation signaling in mesenchymal stem cells. PBM-induced activation of cytochrome c oxidase enhances mitochondrial function, leading to increased ATP synthesis, controlled ROS production, and modulation of intracellular calcium signaling. These bioenergetic and metabolic changes act as upstream regulators of transcriptional programs that govern MSC fate decisions. Depending on the cellular context and irradiation parameters, PBM-mediated mitochondrial signaling may favor osteogenic, chondrogenic, angiogenic, or adipogenic differentiation, thereby contributing to tissue regeneration and repair

Recent advances in regenerative dentistry increasingly support the concept that photobiomodulation acts as a biological modulator of MSC behavior rather than as a simple stimulant of tissue repair. Beyond classical microenvironmental cues and lineage-specific transcription factors, accumulating evidence indicates that mitochondrial function represents a central regulatory axis linking laser parameters to MSC fate decisions (Fig. 2). In this context, PBM-induced modulation of mitochondrial metabolism, redox balance, and intracellular signaling has emerged as a unifying mechanism underlying tissue-specific differentiation outcome.

### Osteogenic differentiation and mitochondrial-mediated bioenergetics

As summarized in Table 1, osteogenic differentiation remains the most extensively investigated outcome of photobiomodulation-MSC interactions. Experimental models using SCAPs and DPSCs consistently demonstrate enhanced bone formation following laser irradiation at red or near-infrared wavelengths [16–19]. In vivo studies (Table 1), report increased bone formation when Diode LLLT is combined with ADMSC transplantation, reinforcing the translational relevance of this approach [20].

Recent studies suggest that these osteogenic effects are closely associated with mitochondrial remodeling and metabolic activation. In dental pulp-derived MSCs, photobiomodulation induced ultrastructural changes in mitochondria, endoplasmic reticulum, and autophagic vesicles, accompanied by increased mineral deposition and upregulation of osteogenic markers, including RUNX2, COL1A1, ALP, and BMPs [17]. These changes were dose-dependent and correlated with enhanced secretion of pro-osteogenic cytokines such as IL−6 and IL−8, highlighting mitochondria as bioenergetic and signaling hubs rather than passive energy suppliers.

At the signaling level, evidence indicates that osteogenic commitment under PBM is not driven by a single pathway but by synergistic crosstalk between Wnt/β-catenin and BMP/Smad signaling. Lasers at 660 nm and 980 nm were shown to upregulate RUNX2 and ALP by simultaneously stabilizing β-catenin and promoting phosphorylation of Smad1/5/9; inhibition of either pathway abolished the mineralizing effects of laser irradiation [18, 19, 21, 22]. These findings support the notion that mitochondrial-derived ROS act as second messengers coordinating convergent osteogenic pathways rather than as nonspecific byproducts of increased metabolism.

### Chondrogenic differentiation and wavelength-dependent mitochondrial signaling

Chondrogenic differentiation has been comparatively less explored but presents compelling evidence of wavelength-specific photobiomodulation effects, as detailed in Table 2. Studies using periodontal ligament-derived MSCs demonstrated that photobiomodulation at 808 nm applied on the day of cell seeding is feasible and influences early differentiation events [12].

Importantly, wavelength-dependent mitochondrial activation appears to play a decisive role in these outcomes. Experimental comparisons across infrared, red, green, and blue wavelengths revealed that green laser irradiation (532 nm) inhibited cell proliferation while stimulating chondrogenic differentiation, whereas shorter wavelengths exerted inhibitory or cytotoxic effects under certain conditions [23]. In Table 2 more recent evidence using organic light-emitting diodes tuned to cytochrome c oxidase (CCO) absorption peaks demonstrated that finely tuned red light stimulation enhanced collagen type II expression and glycosaminoglycan deposition, while combined red/NIR exposure favored proliferation without chondrogenic commitment [24–26]. These findings emphasize that controlled mitochondrial activation, rather than maximal bioenergetic stimulation, is critical for directing MSCs toward cartilage lineage.

### Angiogenic differentiation, migration, and mitochondrial ROS signaling

Angiogenesis is a prerequisite for successful tissue regeneration and is strongly influenced by MSC paracrine activity. As shown in Table 3, photobiomodulation enhances angiogenic differentiation and trophic factor secretion across multiple MSC sources, including PDLSCs, HUVECs and BMSCs [27–29]. Two consecutive photobiomodulation applications at 660 nm and 2.4 J/cm² were shown to produce superior angiogenic outcomes compared with higher single doses, reinforcing the importance of dose optimization [30].

Mechanistically, mitochondrial ROS signaling plays a central role in photobiomodulation-induced angiogenic and migratory responses. Rather than directly driving endothelial differentiation, photobiomodulation appears to modulate the MSC secretome, enhancing the release of pro-angiogenic factors and promoting greater cell survival, particularly under ischemic or inflammatory microenvironmental conditions.

### Adipogenic differentiation and metabolic plasticity

Adipogenic differentiation remains one of the most debated aspects of photobiomodulation-MSC interactions. In Dentistry, adipose tissue has been associated with patients with periodontitis, considering the therapeutic potential of adipose-derived cells in promoting bone neoformation in cases of severe periodontitis [31]. 

Although the formation of adipose tissue in the oral cavity is not a widely explored phenomenon in the dental literature, it is essential to investigate cellular differentiation processes under specific experimental conditions, particularly under laser irradiation. This approach enables the analysis of changes in gene regulatory mechanisms, transcription factors, and cellular signaling pathways involved in tissue differentiation.

In this context, the study of laser application in different oral cavity–derived cell populations, with an emphasis on adipogenesis, represents a relevant strategy for understanding the cellular and molecular mechanisms involved in adipose tissue formation, contributing to the advancement of regenerative therapies in Dentistry.

As summarized in Table 4, Diode low-energy laser irradiation at 660 nm increased proliferation and adipogenic differentiation of adipose-derived MSCs, particularly at doses around 1.0 J/cm², with effects becoming evident at 48 - and 72-hours post-irradiation [32]. Similar proliferative responses were observed with Ga-Al-As lasers at 830 nm [33], while Nd: YAG–based PBM enhanced the immunomodulatory potential of ADSCs in inflammatory disease models [34].

Recent metabolic studies provide insight into these divergent outcomes. Exposure of adipose-derived MSCs to red (660 nm) and near-infrared (810 nm) light preserved mitochondrial metabolic activity and supported migration and regenerative potential, whereas blue light (455 nm) induced excessive ROS production, resulting in reduced proliferation and impaired adipogenic differentiation [38, 39]. These findings reinforce the concept of a hermetic mitochondrial response, in which controlled ROS signaling supports differentiation and metabolic plasticity, while excessive oxidative stress compromise’s cellular function.

Adipogenesis is often described as a central regulatory axis, particularly due to the increase in mitochondrial biogenesis, respiratory chain activity, and ATP production observed during differentiation [35]. However, this response is far from uniform or linear. Evidence suggests that mitochondrial alterations can both promote and redirect cell fate, depending on multiple experimental and contextual factors. Parameters such as the basal metabolic state of the cells, the stage of differentiation, substrate availability, and the intensity of oxidative stress play a decisive role.

Notably, moderate levels of reactive oxygen species (ROS) may act as pro-adipogenic signals, whereas excessive ROS levels tend to trigger stress responses, impair differentiation, or shift lineage commitment toward alternative phenotypes [36]. In addition, variations in stimulation parameters, such as dose, exposure time, and wavelength in photobiomodulation studies, can differentially modulate mitochondrial dynamics, affecting processes including fusion/fission balance, membrane potential, and redox signaling. Therefore, although mitochondria are key players in adipogenesis, their role should not be interpreted as deterministic, but rather as part of a highly responsive network in which subtle changes in experimental conditions can significantly influence lineage outcomes.

### Integrative perspective: mitochondria as a unifying regulatory axis

The studies summarized in Tables 1, 2, 3 and 4 demonstrate that photobiomodulation does not act as a universal enhancer of MSC differentiation but rather as a context-dependent modulator of cellular behavior. Mitochondrial bioenergetics link laser parameters to lineage-specific outcomes by acting as metabolic checkpoints that integrate ATP production and redox signaling. Furthermore, PBM has shown the capacity to rescue mitochondrial dysfunction under adverse conditions.

Quantum hyperlight exposure significantly improved mitochondrial activity, proliferation, and survival of Wharton’s jelly-derived MSCs cultured under high-glucose stress, highlighting the therapeutic potential of mitochondrial-targeted photobiomodulation in compromised microenvironments [37]. From a narrative standpoint, recent advances indicate that successful translation of photobiomodulation-MSC therapies will depend on moving beyond descriptive outcomes toward mechanistically informed and parameter-sensitive approaches. Understanding how mitochondrial function integrates photobiomodulation signals with MSC fate decisions is essential for optimizing regenerative strategies in dentistry and related fields.

**Table 1:** List of in vitro osteogenic studies

Reference	Author	Year	Source of MSCs	PBM type	Wavelength (nm)	Energy density	Model	Differentiation	Control group	Outcome
[20]	Rahmati A et al.	2022	SCAPs	LED + Diode Laser	640/940 nm	4 J/cm²	In vitro	Osteogenic and Odontogenic.	Non-irradiated	Enhance osteogenic differentiation and cell viability.
[17]	Chen J et al.	2022	DPSCs	LED	640 nm	2–10 J/cm²	In vitro	Osteogenic.	Non-irradiated	TRPV1-mediated enhances osteogenic differentiation.
[21]	Rezaei et al.	2025	DPSCs	Diode Laser	660 nm	3 J/cm²	In vitro	Odontogenic and osteogenic.	Non-irradiated	Enhance DSP secretion, VEGF, proliferation.
[18]	Rahmati A et al.	2022	SCAPs	LED	640 nm	4 J/cm²	In vitro	Osteogenic.	Non-irradiated	Increased ALP activity and enhanced differentiation.
[19]	El-Din et al.	2024	MSCs	Diode Laser	660 to 810 nm (range studied)	2–6 J/cm²	In vitro	Osteogenic.	Non-irradiated	Increase of RUNX2, OCN and mineralized nodules.
[16]	Eğlenen M et al.	2023	ADMSCs	Diode Laser	970 nm	8 J/cm²	In vivo	Osteogenic.	No functional appliance	Increased bone formation with Diode Laser and ADMSCs.
[22]	Bai J et al.	2021	BMSCs and HUVECs	Diode Laser	810 to 830 nm	1.8 J/cm²	In vivo	Osteogenic.	Not specified	Increased angiogenesis and osteogenic differentiation.
[38]	Mohaghegh S et al.	2020	BMSCs	Diode Laser	810 to 830 nm	4–6 J/cm²	In vivo	Osteogenic.	Saline solution injection	Increased bone formation and improved wound healing (synergistic effect).

**Table 2:** List of in vivo chondrogenic studies

Reference	Author	Year	MSC source	PBM type	Wavelength (nm)	Energy density	Model	Differentiation	Control group	Outcome
[39]	Mahmoudian A et al.	2024	PDLSCs	Diode Laser PBM	808 nm	4 J/cm²	In vitro	Chondrogenic.	Non-irradiated	Increased early-stage differentiation; effective at seeding day.
[12]	Rigi-Ladez MA et al.	2022	PDLSCs	Diode Laser	810 to 940 nm	0.5–2.5 J/cm²	In vitro	Chondrogenic	Non-irradiated	Cell viability and proliferation were optimal at 940 nm.
[23]	Hakimiha N et al.	2025	PDLSCs	Diode laser PBM	808 nm	3–9 J/cm²	In vitro	Chondrogenic	Non-irradiated	Enhanced proliferation and migration of periodontal cells.
[24]	Tim et al.	2021	MSCs	Diode Laser	808 nm	0.8–1.4 J/cm²	In vivo	Chondrocyte	OA control	Enhanced COL2, aggrecan, lowered IL−1β.
[25]	Xiang et al.	2020	MSCs	Diode Laser	660 to 904 nm	1–5 J/cm²	In vivo	Chondrocyte	No PBM	Improved histological cartilage scores.
[26]	de Oliveira et al.	2017	hESCs	Diode Laser	808 nm	4 J per point;	In vivo	Chondrocyte	OA without PBM.	Increased TNF-α; Reduced inflammation and pain.

**Table 3:** List of in vivo angiogenic studies

Reference	Author	Year	MSC source	PBM Type	Wavelength (nm)	Energy density	Model	Differentiation	Control group	Outcome
[27]	Atarbash Mogh adam F et al.	2024	hPDLSCs	Diode Laser	660 nm	2.4–3.9 J/cm²	In vitro	Angiogenic.	Non-irradiated cells	Enhance angiogenesis, being 2.4 J/cm² the most effective parameter.
[28]	Seo I et al.	2023	hMSCs	LED	610 nm	3.5 V; 65 mA; 115.4 Cd/m²	In vitro	Angiogenic.	Non-treated group	Increased VEGF, HGF, FGF2 expression.
[29]	Kim K et al.	2019	HUCB - MSCs	LED	633 nm	1.65 to 7.12 mW/cm²	In vitro	Angiogenic.	Non-irradiated group	Increased VEGF, ANGPT1/2, PDGF; Decreased apoptosis.
[30]	Sarveazad A et al.	2019	hADSCs	Diode Laser	660 nm	100 mW/cm²	In vitro	Angiogenic + myogenic differentiation	No intervention group	Increased angiogenesis and tissue regeneration.
[40]	Bai J et al.	2021	BMSCs + HUVECs	GaAlAs LLL.	Non specified	2.4–3.9 J/cm²	In vivo	Angiogenic.	Non specified	Increased type H vessels; ROS/HIF−1α pathway; Enhanced osteogenesis with angiogenesis.
[41]	Sarveazad A et al.	2019	hADSCs	Diode Laser	660 nm	3.5 V; 65 mA; 115.4 Cd/m²	In vivo	Angiogenic.	Non-treated group	Increased angiogenesis and enhanced tissue regeneration.
[42]	Pires-Oliveira et al.	2010	BMSCs	GaAIAs laser	904 nm	50 mJ/cm²	In vivo	Angiogenic and osteogenic.	Non-irradiated.	Accelerated bone repair; enhanced early tissue regeneration; indirect angiogenic stimulation.

**Table 4:** List of vitro adipogenic studies

Reference	Author	Year	MSC source	PBM type	Wavelength (nm)	Energy density	Model	Differentiation	Control group	Outcome
[32]	Barboza C et al.	2014	hMSCs/hADSCs	Diode Laser	660 nm	0.5–1.0 J/cm²	In vitro	Adipogenic	Non-irradiated cells	hADSCs showed higher sensitivity and increased proliferation.
[33]	Min K et al.	2015	hADSCs	GaAlAs + Diode Laser	830 nm	Not specified	In vitro	Adipogenic	Non-irradiated group	Increased proliferation of irradiated ADSCs.
[43]	Fernandes A et al.	2019	hMSCs	LED	670 nm	20 mJ/cm²	In vitro	Adipogenic.	Non-irradiated group	PBM induced adipogenic differentiation via photobiomodulation signaling.
[34]	He L et al.	2023	hADSCs	Nd: YAG laser	1064 nm	Pulsed irradiation (1 s cycles) nm	In vitro	Adipogenic + immunomodulatory response	Non-irradiated ADSCs	Increased anti-inflammatory response and immunomodulation.

Current evidence regarding PBM-induced adipogenic differentiation remains limited and is predominantly derived from in vitro studies. To date, no in vivo studies investigating adipogenic differentiation in dental applications have been reported [44].

#### Microenvironmental, metabolic, and mitochondrial determinants of MSC lineage commitment

These cues regulate lineage-specific transcription factors and modulate intracellular metabolism and mitochondrial function, influencing cell fate. Oxygen tension, nutrient availability, extracellular matrix composition, and mechanical forces collectively determine mitochondrial bioenergetics, redox balance, and signaling pathways that control whether MSCs remain quiescent, proliferate, migrate, or commit to a specific lineage.

Consistent with this concept, MSCs tend to differentiate more efficiently into tissues resembling their environment of origin. Bone marrow-derived MSCs (BM-MSCs), for instance, demonstrate a greater propensity for osteogenic differentiation, whereas adipose-derived MSCs favor adipogenic pathways. Factors such as vascularization, mechanical stimulus (including pressure, tension, and matrix stiffness), and chemical components of the microenvironment, such as pH, cytokines, and growth factors, modulate not only cellular behavior but also mitochondrial metabolism and the cellular response to light-based therapies [45, 46]. These microenvironmental variables influence how MSCs interpret photobiomodulatory signals, reinforcing the concept that photobiomodulation outcomes are context-dependent rather than universally stimulatory.

In bone tissue, the osteogenic microenvironment combined with Diode Laser and LED has been shown to increase the expression of key transcription factors such as Runx2 and Osterix (Osx), accelerating osteoblastic differentiation [37]. From a metabolic perspective, this process is associated with increased mitochondrial activity and controlled redox signaling, which support ATP production and activation of osteogenesis-related pathways.

The in vivo evidence supports the biological relevance of the angiogenic effects observed in laboratory studies (Table 3) [40]. Notably, demonstrated that photobiomodulation (PBM) associated with bone marrow-derived mesenchymal stem cells (BMSCs) and human umbilical vein endothelial cells (HUVECs) increased the formation of type H vessels through activation of the ROS/HIF−1α signaling pathway, promoting both angiogenesis and osteogenesis [40]. Although HUVECs are endothelial cells and not MSCs, their inclusion provides important insights into the vascular microenvironment that supports MSC-mediated tissue regeneration.

The enhanced formation of type H vessels is closely associated with mitochondrial regulation of cell migration, survival, and paracrine signaling, illustrating how PBM-induced vascular responses can indirectly influence MSC behavior and function [31]. These findings highlight the interplay between endothelial and mesenchymal cells during tissue repair and reinforce the importance of adequate vascularization for successful bone regeneration and implant-related therapies [40].

The choice of MSC source further influences tissue–cell correlation by affecting both cell quantity and intrinsic metabolic competence. Adipose tissue provides a high yield of MSCs, making it suitable for applications requiring large cell numbers. Bone marrow-derived MSCs, although less abundant, often exhibit superior proliferative, osteogenic, and angiogenic potential [47]. These qualitative differences may be partially attributed to variations in mitochondrial content, respiratory capacity, and redox adaptability among MSC populations derived from distinct tissues. Consequently, volumetric regenerative procedures may benefit from abundant cell sources, whereas bone and cartilage regeneration demand MSCs with higher functional and metabolic quality.

Taken together, tissue to cell correlation in MSC-based therapies reflects a complex interplay between cellular origin, microenvironmental cues, and intracellular metabolic regulation. Mitochondria emerge as central integrators of these signals, translating physical and biochemical stimuli, including low-level laser irradiation, into lineage-specific responses. Strategic selection of the MSC source, combined with careful modulation of the microenvironment and photobiomodulation parameters, is therefore essential to optimize regenerative outcomes and ensure effective tissue-specific differentiation [48].

It is important to avoid oversimplification that may lead to neglecting tissue-specific metabolic programming and to misleading interpretations of PBM’s capacity to direct stem cell fate, particularly when extrapolating findings from one MSC source to another without accounting for their distinct lineage predispositions and metabolic behaviors [10, 49, 50].

Furthermore, the signaling pathways, transcription factors, and growth factors involved in osteogenic, chondrogenic, adipogenic, and endothelial differentiation are highly interconnected, reinforcing that lineage commitment is a complex, tightly regulated biological process that cannot be interpreted through isolated mechanisms or simplified assumptions, as summarized in the following table (Table 5).

**Table 5:** Summary of the major growth factors, signaling pathways, transcription factors, and molecular crosstalk involved in mesenchymal stem cell (MSC) differentiation toward osteogenic, chondrogenic, adipogenic, and endothelial lineages, highlighting the principal ligands and regulatory pathways governing bone, cartilage, adipose, and vascular endothelial tissue formation

Lineage / tissue	Growth factors	Major signaling pathways	Key transcription factors	Main crosstalk with other lineages
Osteogenic (Bone Tissue)	BMPs, FGF2	Wnt/β-catenin, BMP/Smad, Notch	RUNX2	Shares BMP/Smad, Wnt/β-catenin, and Notch signaling with other MSC differentiation pathways.
Chondrogenic (Cartilage Tissue)	HGF	Hedgehog/GLI	SOX9, RUNX1	Closely interacts with osteogenesis through RUNX1 and Hedgehog signaling.
Adipogenic (Adipose Tissue)	IGF-1, Insulin, BMP4, BMP7, FGF1, FGF2	PI3K/Akt, Insulin/IGF-1, BMP/Smad	PPARγ, C/EBPα	PPARγ antagonizes RUNX2-mediated osteogenesis.
Endothelial (Angiogenic) Lineage	VEGF, PDGF, FGF2, Angiopoietin-1/2	VEGF/VEGFR, PI3K/Akt, MAPK/ERK	HIF-1α, ETS-1, ERG	VEGF and FGF2 coordinate vascularization and bone regeneration.

## Biological limits, mitochondrial redox balance, and the need for parameter standardization

Despite growing evidence supporting the combined use of LLLT and MSCs, relevant biological and methodological challenges remain. A key limitation is the control of intracellular oxidative balance. Osteogenic differentiation is highly sensitive to redox status, and excessive reactive oxygen species (ROS) may disrupt early differentiation, impair matrix mineralization, and compromise tissue formation [40]. Mitochondrial function plays a central role, regulating both ATP production and ROS signaling, essential for lineage commitment but harmful when dysregulated.

Key transcription factors, RUNX2 for osteogenesis [19], Sox9 for chondrogenesis [24] and PPARy for adipogenic and myogenic pathways [43], are closely linked to cellular metabolism. Mitochondrial bioenergetics and redox signaling influence their activity, reinforcing the link between transcriptional regulation and metabolic control. Variations in mitochondrial responsiveness among MSCs from different tissues may partly explain the heterogeneity observed after photobiomodulation.

Another important challenge lies in the lack of standardization of laser parameters. Variables such as wavelength, power, energy density, exposure time, and irradiation frequency directly influence mitochondrial responses and mesenchymal stem cell (MSC) behavior. While appropriate doses may promote regenerative effects, excessive or improperly applied irradiation can lead to mitochondrial dysfunction, oxidative stress, and even necrosis [13]. This biphasic response underscores the need for clearly defined therapeutic windows.

Finally, most studies are preclinical and heterogeneous in design, limiting comparability and clinical translation. Addressing these issues requires an integrated approach that considers both molecular markers of differentiation and mitochondrial health as key determinants of regenerative success.

## MSC-based photobiomodulation protocols in regenerative dentistry

The integration of mesenchymal stem cells (MSCs) with light-based therapies has been increasingly reported in the context of regenerative medicine and dentistry. The literature suggests that this combined approach may be associated with improved postoperative healing, as well as functional and aesthetic outcomes, while also providing a potential strategy for modulating MSC behavior in a non-invasive and spatially controlled manner. Nevertheless, the available evidence indicates that further studies are required to better characterize the underlying biological mechanisms involved, particularly those related to cellular metabolism, including mitochondrial bioenergetics, redox signaling, and metabolic plasticity.

Across the included studies, a recurrent limitation was the lack of standardized irradiation protocols. Future investigations would benefit from the development of more consistent experimental and clinical parameters, adapted to specific MSC sources, tissue targets, and clinical applications. In addition, the inclusion of mitochondrial-related outcome measures, such as ATP production, mitochondrial membrane potential, and reactive oxygen species (ROS) dynamics, has been reported in experimental studies but remains inconsistently applied across the literature. Standardization of these parameters may improve comparability between studies and support more robust interpretation of regenerative outcomes. Comparative analyses involving MSCs derived from different tissue sources under similar photobiomodulation conditions are also still limited, although they are considered relevant to clarify potential variability in cellular responses.

Clinical and preclinical syntheses included in this review indicate that, despite promising findings, heterogeneity in laser parameters continues to limit the development of evidence-based clinical protocols. Evidence from peri-implantitis-related applications further supports the need for improved alignment between clinical practice and mechanistic research focused on cellular metabolism and mitochondrial-related responses [11].

This scoping review mapped and synthesized the available literature on the effects of different laser-based therapies on stromal cells, with emphasis on osteogenic, chondrogenic, angiogenic, and adipogenic outcomes. Overall, the studies included suggest that mitochondrial-related processes may be involved in the cellular responses observed following photobiomodulation; however, the heterogeneity of experimental designs and outcome measures limits definitive conclusions regarding the extent and specificity of these relationships.

Further interdisciplinary research involving clinicians, biologists, and bioengineers is required to improve methodological consistency and support the translation of preclinical findings into clinical applications. From a scoping perspective, the current evidence highlights both the potential relevance of MSC–photobiomodulation interactions and the need for standardized methodologies to support future systematic and mechanistic investigations.

## Limitations

This scoping review has some limitations that should be considered. Most of the available evidence is derived from in vitro and animal studies, limiting the direct translation of findings to clinical practice. In addition, substantial heterogeneity was observed regarding stem cell sources, photobiomodulation parameters, and experimental designs, making comparisons between studies difficult. The lack of standardized irradiation protocols remains a major challenge, as variations in wavelength, energy density, and exposure time may significantly influence biological outcomes. Furthermore, evidence regarding adipogenic differentiation in dentistry, particularly from in vivo studies, is still scarce. Finally, differences between dental-derived and non-dental mesenchymal stem cells may affect the interpretation and generalization of the reported findings. 

## Data Availability

No datasets were generated or analysed during the current study.

## References

[1] Minervini G, Franco R, Marrapodi MM, Mehta V, Fiorillo L, Badnjević A, Cervino G, Cicciù M (2023) The Association between COVID-19 Related Anxiety, Stress, Depression, Temporomandibular Disorders, and Headaches from Childhood to Adulthood: A Systematic Review. Brain Sci. 10.3390/brainsci13030481 36979291 PMC10046052

[2] Shi X, Mao J, Liu Y (2020) Pulp stem cells derived from human permanent and deciduous teeth: Biological characteristics and therapeutic applications. Stem Cells Transl Med. 10.1002/sctm.19-0398 31943813 PMC7103623

[3] Dominici M, Le Blanc K, Mueller I et al (2006) Minimal criteria for defining multipotent mesenchymal stromal cells. The International Society for Cellular Therapy position statement. Cytotherapy 8(4):315–317. 10.1080/14653240600855905 16923606

[4] Lazăr L, Manu DR, Dako T, Mârțu MA, Suciu M, Ormenișan A, Păcurar M, Lazăr AP (2022) Effects of Laser Application on Alveolar Bone Mesenchymal Stem Cells and Osteoblasts: An In Vitro Study. Diagnostics (Basel) 12(10):2358. 10.3390/diagnostics12102358 36292047 PMC9600660

[5] Chung H, Dai T, Sharma SK, Huang YY, Carroll JD, Hamblin MR (2012) The nuts and bolts of low-level laser (light) therapy. Ann Biomed Eng 40(2):516–533. 10.1007/s10439-011-0454-7 22045511 PMC3288797

[6] Ankrum JA, Ong JF, Karp JM (2014) Mesenchymal stem cells: immune evasive, not immune privileged. Nat Biotechnol 32(3):252–60. 10.1038/nbt.2816 24561556 PMC4320647

[7] Uccelli A, Moretta L, Pistoia V (2008) Mesenchymal stem cells in health and disease. Nat Rev Immunol 8(9):726–736. 10.1038/nri2395 19172693

[8] Zakrzewski W, Dobrzyński M, Szymonowicz M, Rybak Z (2019) Stem cells: past, present, and future. Stem Cell Res Ther 10(1):68. 10.1186/s13287-019-1165-5 30808416 PMC6390367

[9] Purbhoo-Makan M, Houreld NN, Enwemeka CS (2022) The effects of blue light on human fibroblasts and diabetic wound healing. Life (Basel) 14(9):1431. 10.3390/life12091431 PMC9505688 36143466

[10] Ullah I, Subbarao RB, Rho GJ (2015) Human mesenchymal stem cells—current trends and future prospects. Biosci Rep 35(2):e00191. 10.1042/BSR20150025 25797907 PMC4413017

[11] Mojaver S, Fiorellini J, Sarmiento H (2025) Advancing peri-implantitis treatment: a scoping review of breakthroughs in implantoplasty and Er:YAG laser therapies. Clin Exp Dent Res. 10.1002/cre2.70104 40071719 PMC11898008

[12] Wang T, Ye J, Zhang Y, Li J, Yang T, Wang Y, Jiang X, Yao Q (2024) Role of oxytocin in bone. Front Endocrinol (Lausanne) 15:1450007. 10.3389/fendo.2024.1450007 39290327 PMC11405241

[13] de Freitas LF, Hamblin MR (2016) Proposed mechanisms of photobiomodulation or low-level light therapy. IEEE J Sel Top Quantum Electron 22(3):7000417. 10.1109/JSTQE.2016.2561201 28070154 PMC5215870

[14] Wang L, Wu F, Liu C et al (2019) Low-level laser irradiation modulates the proliferation and the osteogenic differentiation of bone marrow mesenchymal stem cells under healthy and inflammatory conditions. Lasers Med Sci 34(1):169–178. 10.1007/s10103-018-2673-8 30456535

[15] Dang Y, Ye X, Weng Y, Tong Z, Ren Q (2010) Effects of the 532-nm and 1,064-nm Q-switched Nd:YAG lasers on collagen turnover of cultured human skin fibroblasts: a comparative study. Lasers Med Sci 25(5):719–726. 10.1007/s10103-009-0657-4 20490593

[16] Eğlenen MN, Tuğlu Mİ, Aydemir I, Güleç A (2023) The Condylar Effects of Mesenchymal Stem Cells, Low-Level Laser Therapy and Grape Seed Extract on Functional Mandibular Advancement of the Rat Mandible. Turk J Orthod 36(2):79–86. 10.4274/TurkJOrthod.2022.2022.92 37345991 PMC10318849

[17] Chen J, Sang Y, Li J, Zhao T, Liu B, Xie S, Sun W (2022) Low-level controllable blue LEDs irradiation enhances human dental pulp stem cells osteogenic differentiation via transient receptor potential vanilloid 1. J Photochem Photobiol B. 10.1016/j.jphotobiol.2022.112472 35660312

[18] Rahmati A, Abbasi R, Najafi R et al (2022) Effect of diode low level laser and red light emitting diode irradiation on cell proliferation and osteogenic/odontogenic differentiation of stem cells from the apical papilla. BMC Oral Health. 10.1186/s12903-022-02574-8 36434589 PMC9701043

[19] Chen Y, Li Z, Zhou M et al (2022) A comparative analysis of the osteogenic capacity of osteoblasts from newborn and two-week-old rats. Acta Histochem 124:151858. n. 2 10.1016/j.acthis.2022.151858 35121379

[20] Rahmati A, Abbasi R, Najafi R et al (2022) Effect of diode low level laser and red light emitting diode irradiation on cell proliferation and osteogenic/odontogenic differentiation of stem cells from the apical papilla. BMC Oral Health. 10.1186/s12903-022-02574-8 36434589 PMC9701043

[21] Rezaei F, Shakoori S, Fazlyab M et al (2025) Effect of low-level laser on proliferation, angiogenic and dentinogenic differentiation of human dental pulp stem cells. BMC Oral Health 25:441. 10.1186/s12903-025-05656-5 40148901 PMC11948823

[22] El-Din MS, El-Sharkawy A, Abdelrahman H et al (2024) Different wavelengths of laser: are they significant for treatment of denture stomatitis? an in-vitro study. BMC Oral Health 24:71. 10.1186/s12903-023-03845-8 38212756 PMC10782685

[23] Etemadi A, Aghaie M, Sayar F, Chiniforush N (2024) Effect of photobiomodulation therapy with 660 and 980 nm diode lasers on differentiation of periodontal ligament mesenchymal stem cells. Sci Rep 14(1):20587. 10.1038/s41598-024-71386-3 39232133 PMC11375153

[24] Rigi-Ladez MA, Hendi SS, Mirzaei A, Gholami L, Fekrazad R (2022) Near Infrared Laser Photobiomodulation of Periodontal Ligament Stem Cells. Chin J Dent Res. ;25(1):57–65. 10.3290/j.cjdr.b2752657. PMID: 35293711 35293711

[25] Hanane Pourheidary a,Maryam Tehranchi a Ferial Taleghani a,Mahshid Hodjat b, Maryam Pourhajibagher b, Hakimiha N. Evaluation of the combined effects of photobiomodulation therapy and periodontal ligament stem cell-derived exosomes on the proliferation and migration of human gingival fibroblasts: An in vitro study. 10.1016/j.jdent.2025.106053 40850464

[26] Atarbashi-Moghadam F, Samadi-Rad A, Hakimiha N, Taghipour N, Mahmoudian A, Azadi A, Nokhbatolfoghahaei H (2024) The impact of photobiomodulation on angiogenic differentiation of two different dental derived stem cells using two irradiation protocols: an in vitro investigation. BMC Oral Health 24(1):1090. 10.1186/s12903-024-04753-1 39277707 PMC11402196

[27] Seo I, Kim SW, Hyun J, Kim YJ, Park HS, Yoon JK, Bhang SH (2023) Enhancing viability and angiogenic efficacy of mesenchymal stem cells via HSP90α and HSP27 regulation based on ROS stimulation for wound healing. Bioeng Transl Med 8(5):e10560. 10.1002/btm2.10560 37693062 PMC10487335

[28] Seo I, Kim S-W, Hyun J et al (2023) Enhancing viability and angiogenic efficacy of mesenchymal stem cells via HSP90α and HSP27 regulation based on ROS stimulation for wound healing. Bioeng Transl Med 8(5):e10560. 10.1002/btm2.10560 37693062 PMC10487335

[29] Sarveazad A, Babahajian A, Yari A, Rayner CK, Mokhtare M, Babaei-Ghazani A, Agah S, Mahjoubi B, Shamseddin J, Yousefifard M (2019) Combination of laser and human adipose-derived stem cells in repair of rabbit anal sphincter injury: a new therapeutic approach. Stem Cell Res Ther 10(1):367. 10.1186/s13287-019-1477-5 31791407 PMC6889595

[30] ‌Fekrazad R, Asefi S, Eslaminejad MB, Taghiar L, Bordbar S, Hamblin MR (2019) Photobiomodulation with single and combination laser wavelengths on bone marrow mesenchymal stem cells: proliferation and differentiation to bone or cartilage. Lasers Med Sci 34(1):115–126. 10.1007/s10103-018-2620-8 30264177 PMC6344244

[31] Bai J, Li L, Kou N, Bai Y, Zhang Y, Lu Y, Gao L, Wang F (2021) Low level laser therapy promotes bone regeneration by coupling angiogenesis and osteogenesis. Stem Cell Res Ther 12(1):432. 10.1186/s13287-021-02493-5 34344474 PMC8330075

[32] Barboza CA, Ginani F, Soares DM, Henriques AC, Freitas Rde A (2014) Low-level laser irradiation induces in vitro proliferation of mesenchymal stem cells. Einstein (Sao Paulo) 12(1):75–81. 10.1590/s1679-45082014ao2824 24728250 PMC4898243

[33] Schieber M, Chandel NS (2014) ROS Function in Redox Signaling and Oxidative Stress. Curr Biol 24(10):R453–R462. https://pmc.ncbi.nlm.nih.gov/articles/PMC4055301/24845678 10.1016/j.cub.2014.03.034 PMC4055301

[34] Min KH, Byun JH, Heo CY, Kim EH, Choi HY, Pak CS (2015) Effect of Low-Level Laser Therapy on Human Adipose-Derived Stem Cells: In Vitro and In Vivo Studies. Aesthetic Plast Surg 39(5):778–782. 10.1007/s00266-015-0524-6 26183254

[35] Castilho-Fernandes A, Lopes TG, Ferreira FU, Rezende N, Silva VF, Primo FL, Fontes AM, Ribeiro-Silva A, Tedesco AC (2019) Adipogenic differentiation of murine bone marrow mesenchymal stem cells induced by visible light via photo- induced biomodulation. Photodiagnosis Photodyn Ther 25:119–127. 10.1016/j.pdpdt.2018.11.013 30458313

[36] He L, Zheng Y, Liu M et al (2023) Nd:YAG-photobiomodulation enhanced ADSCs multilineage differentiation and immunomodulation potentials. Lasers Med Sci 38:190. 10.1007/s10103-023-03818-x 37608016 PMC10444653

[37] Firoozi P, Amiri MA, Soghli N, Farshidfar N, Hakimiha N, Fekrazad R (2024) The role of photobiomodulation on dental-derived stem cells in regenerative dentistry: a comprehensive systematic review. Curr Stem Cell Res Ther 19(4):559–586. 10.2174/1574888x17666220810141411 35950251

[38] Bai J, Li L, Kou N, Bai Y, Zhang Y, Lu Y, Gao L, Wang F (2021) Low level laser therapy promotes bone regeneration by coupling angiogenesis and osteogenesis. Stem Cell Res Ther 12(1):432. 10.1186/s13287-021-02493-5 34344474 PMC8330075

[39] Mohaghegh S, Mohammad-Rahimi H, Eslamian L, Ebadifar A, Badiee MR, Farahani M, Mohebbi Rad M, Motamedian SR (2020) Effect of mesenchymal stem cells injection and low-level laser therapy on bone formation after rapid maxillary expansion: an animal study. Am J Stem Cells 9(5):78–88 33489465 PMC7811931

[40] ‌Lucke LD, Bortolazzo FO, Theodoro V, Fujii L, Bombeiro AL, Felonato M, Dalia RA, Carneiro GD, Cartarozzi LP, Vicente CP, Oliveira ALR, Mendonça FAS, Esquisatto MAM, Pimentel ER, de Aro AA (2019) Low-level laser and adipose-derived stem cells altered remodelling genes expression and improved collagen reorganization during tendon repair. Cell Prolif 52(3):e12580. 10.1111/cpr.12580 30734394 PMC6536450

[41] Tormos KV, Anso E, Hamanaka RB et al (2011) Mitochondrial Complex III ROS Regulate Adipocyte Differentiation. Cell Metabol 14(4):537–544. 10.1016/j.cmet.2011.08.007 PMC3190168 21982713

[42] Lepper C, Fan CM (2010) Inducible lineage tracing of Pax7-descendant cells reveals embryonic origin of adult satellite cells. Genesis 48(7):424–436. 10.1002/dvg.20630 20641127 PMC3113517

[43] Bölükbaşı Ateş G, Ak A, Garipcan B et al (2020) Photobiomodulation effects on osteogenic differentiation of adipose-derived stem cells. Cytotechnology 72:247–258. 10.1007/s10616-020-00374-y 32016710 PMC7192995

[44] Sarveazad A, Janzadeh A, Taheripak G, Dameni S, Yousefifard M, Nasirinezhad F (2019) Co-administration of human adipose-derived stem cells and low-level laser to alleviate neuropathic pain after experimental spinal cord injury. Stem Cell Res Ther 10(1):183. 10.1186/s13287-019-1269-y 31234929 PMC6591829

[45] Apte RS, Chen DS, Ferrara N (2019) VEGF in Signaling and Disease: Beyond Discovery and Development. Cell 176(6):1248–1264. 10.1016/j.cell.2019.01.021 30849371 PMC6410740

[46] Matsumoto K, Funakoshi H, Takahashi H, Sakai K (2014) HGF-Met Pathway in Regeneration and Drug Discovery. Biomedicines 2(4):275–300. 10.3390/biomedicines2040275 28548072 PMC5344275

[47] Sarveazad A, Babahajian A, Yari A, Rayner CK, Mokhtare M, Babaei-Ghazani A, Agah S, Mahjoubi B, Shamseddin J, Yousefifard M (2019) Combination of laser and human adipose-derived stem cells in repair of rabbit anal sphincter injury: a new therapeutic approach. Stem Cell Res Ther 2(1):367. 10.1186/s13287-019-1477-5 PMC6889595 31791407

[48] Bölükbaşı Ateş G, Ak A, Garipcan B et al (2020) Photobiomodulation effects on osteogenic differentiation of adipose-derived stem cells. Cytotechnology 72:247–258. 10.1007/s10616-020-00374-y 32016710 PMC7192995

[49] Rocha EA, Alvarez MMP, Pelosine AM, Carrilho MRO, Tersariol ILS, Nascimento FD (2022) Laser Photobiomodulation 808 nm: Effects on Gene Expression in Inflammatory and Osteogenic Biomarkers in Human Dental Pulp Stem Cells. Front Pharmacol 12:782095. 10.3389/fphar.2021.782095 35111053 PMC8802107

[50] Sridharan K, Waheed TO, Staehlke S et al (2025) Light exposure as a tool to enhance the regenerative potential of adipose-derived mesenchymal stem/stromal cells. Cells 14(15):1143. 10.3390/cells14151143 40801576 PMC12346127

